# Evaluation of the implementation of hospital hygiene components in 30 health-care facilities in the autonomous district of Abidjan (Cote d’Ivoire) with the WHO Infection Prevention and Control Assessment Framework (IPCAF)

**DOI:** 10.1186/s12913-023-09853-2

**Published:** 2023-08-16

**Authors:** Doumbia Mariamou Cissé, Essis Esme Marie Laure, Koné Atioumounan Blaise, N’gbesso N’gbesso Jean Paul, Mbengue Valérie Gbonon, Cissé Raïssa Adja Mayaka, Gagne Doh Eugénie, Dagnan N’cho Simplice, Kouadio Luc Philippe, Samba Mamadou

**Affiliations:** 1 Minister of Health, Public Hygiene and Universal Health Coverage of Directorate General of Health, BP V 4 Abidjan, CI-AB. IV93. 14/ CI.AB. 110, Abidjan, Côte d’Ivoire; 2 Department of Public Health, Felix Houphouët Boigny University, UFR of Pharmaceutical and Biological Sciences, 22 BP 582 Abidjan 22, Abidjan, Côte d’Ivoire; 3 National Institute of Public Health, BPV 47 Abidjan, CI-AB. IV93. 14/ CI.AB. 110, Abidjan, Côte d’Ivoire; 4 Reproductive Health Research Unit of Cote d’Ivoire, BPV 47 Abidjan, CI-AB. IV93. 14/ CI.AB. 110, Abidjan, Côte d’Ivoire; 5 National Institute of Public Hygiene, BPV 14 Abidjan 01, CI-AB. IV93. 14/ CI.AB. 110, Abidjan, Côte d’Ivoire; 6 Biology and Health Laboratory, University of Félix Houphouët Boigny, 22 B.P. 582, Abidjan 22, CI-AB. IV93. 14/ CI.AB. 110, Abidjan, Côte d’Ivoire; 7 Pasteur Institute of Cote d’Ivoire, 01 BP 490 Abidjan 01, CI-AB. IV93. 14/ CI.AB. 110, Abidjan, Côte d’Ivoire; 8 Molecular Genetics Platform of the National Reference Center, CI-AB. IV93. 14/ CI.AB. 110, Abidjan, Côte d’Ivoire; 9 Directorate of Public Hygiene and Health-Environment, BP V 4 Abidjan, CI-AB. IV93. 14/ CI.AB. 110, Abidjan, Côte d’Ivoire; 10 Department of Public Health and Biostatistics, Félix Houphouët Boigny University, UFR of Medical Sciences, 01 BP V34, Abidjan, Réf.ECI554. Abidjan, CI-AB. IV93. 14/ CI.AB. 110, Abidjan, Côte d’Ivoire; 11 Department of Public Health Department, UFR of Odonto- stomatology, Félix Houphouët Boigny University, 01 BPV 34 Abidjan 01, CI-AB. IV93. 14/ CI.AB. 110, Abidjan, Côte d’Ivoire

**Keywords:** Infection Prevention and Control, Survey, Evaluation, Hand Hygiene, Healthcare associated infections, Patient safety, Health-care facilities, Cote d’Ivoire

## Abstract

**Introduction:**

As part of the implementation of its mission “to integrate hygiene activities into healthcare”, the general directorate of health conducted in 2018 with its technical structures, an evaluation of the implementation of Infection Prevention and Control (IPC) using the WHO IPCAF tool in 30 health-care facilities in the autonomous district of Abidjan.

**Materials and methods:**

This were a cross-sectional survey with a conceptualized component considering the issue of injection safety and sanitary waste management, which was conducted in the named health-care facilities from March 20 to 28, 2018. The scores of the essential components of the IPC made it possible to assess the IPC level of each health-care facility evaluated and the overall IPCAF score of all facilities.

**Results:**

The overall median IPCAF score of the health-care facilities was 242.5/800 and corresponded to an inadequate level overall. No facility reached the “advanced” level of performance, 5 facilities (17%) reached the “intermediate” level, 10 (33%) fell into the “basic” level, and 15 (50%) were at the “inadequate” level. Baseline institutions had much higher scores than first contact institutions.

**Conclusion:**

IPC component activities were inadequate and fragmented in the under-resourced health facilities at the time of the assessment. It would be appropriate to provide adequate resources and develop expertise in IPC through strong political will and leadership. This will contribute to the achievement of universal health insurance objectives with safe health services for patients.

## Introduction

Healthcare associated infections contracted during a stay or a passage in a hospital environment constitute a public health problem in the world [1–3] and in particular in Africa [4–7]. They are characterized by a heavy financial impact and mortality due to the virulence of the pathogenic agents and their resistance to the effects of anti-infectious medicine [8–11]. These infections are mainly due to the failure of hygiene mechanisms in health-care facilities [12, 13]. Indeed, hygiene in health-care facilities plays an essential role in the prevention of diseases and in the promotion of the quality of care [6]. Its control is therefore an important step in the search for sustainable solutions to the thorny problem of healthcare associated infections, particularly to the improvement of the living environment of users of health care facilities.

To this end, international guidelines for IPC and hand hygiene have been initiated in health facilities around the world. These guidelines are a key element of WHO’s strategies to prevent current and future threats from infectious diseases (Ebola, Dengue hemorrhagic), strengthen the resilience of health services to cope with large-scale pandemics (covid 19), help combat antimicrobial resistance (AMR) and improve the overall quality of healthcare delivery [14, 15]. The World Health Organization (WHO) has developed tools to assist in their implementation, including the Infection Prevention and Control Assessment Framework (IPCAF) and the Hand Hygiene Self-Assessment Framework (HHSAF), to provide a framework for improvement [8, 16]. However, not all countries are at the same level of implementation of these tools. Although studies have proven the feasibility and effectiveness of these guidelines using the evaluation frameworks proposed by the WHO in several countries around the world [17–20], much remains to be done, especially in developing countries [9, 11, 13, 21].

In Cote d’Ivoire, several actions have been carried out by the Ministry of Health since 2006 (the year the former General Directorate of Public Hygiene was established) with technical and financial support from partners through capacity building activities in equipment and training, awareness raising, advocacy, etc. In addition, to achieve the objectives of the “My Health, My Life” policy, the Ministry in charge of health made a firm commitment to sanitize hospitals in 2017, which was declared the “Year of Hygiene.“ However, healthcare-associated infections are recurrent [10, 22] and are very often associated with noncompliance with hygiene rules by health-care personnel and users [6]. Very little work has been done to assess the level of implementation of international guidelines for IPC in Ivorian health facilities.

A study conducted at the University Hospital Center (UHC) of Bouake, focusing solely on the WHO multimodal hand hygiene improvement strategy, highlighted the need to implement a national strategy to build a robust hygiene system to increase patient safety [10].

With the aim of filling this scientific gap, the present study was conducted to assess the state of implementation of hygiene and IPC in public health facilities to compare the performance of their practice with the essential components of the WHO IPCAF framework adapted to the Ivorian health context.

The results of this work could help in the implementation of good hospital hygiene practices and surveillance of healthcare-associated infections in target health facilities.

## Materials and methods

### Setting of the study

**Fig. 1:**
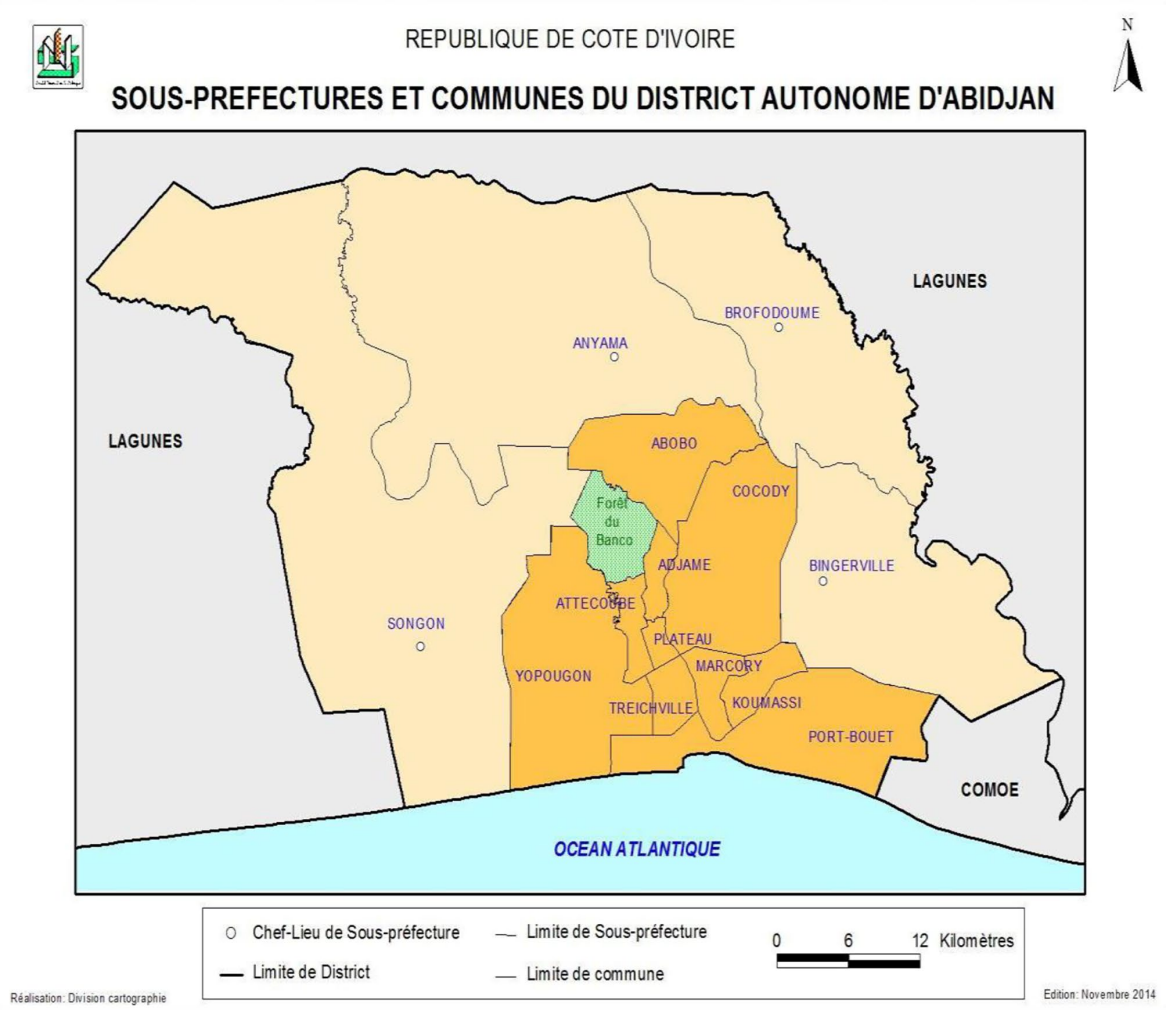
Study area (Autonomous District of Abidjan)

The autonomous district of Abidjan, located in the southeast of the country, is bordered by the Atlantic Ocean and the Ebrié Lagoon. The economic capital of the country is The Autonomous District of Abidjan. As such, it has a special legal personality with financial autonomy *(*Fig. 1*)*. It has ten urban municipalities, four Sous Prefecture *(*Fig. 1*)* with a total population of 4,707,404 (RGPH, 2021). It has.

- two Health Regions (Abidjan-1, Abidjan-2),
- ten Health Districts,
- 636 public health-care facilities, including 04 University Hospital Centers, 09 general hospitals and 623 first contact health-care facilities (ESPC).

### Type of study

It is a normative evaluation. The standard is a synthesis of criteria established by the WHO on IPC and a consensus of national experts (**1**. the Head of Studies for Quality, Hygiene and Safety of Care at the General Health Directorate, **2**. the Head of the Hygiene Promotion Department at the DHPSE, **3**. a Hygiene and Sanitation Technician at the Sub-Directorate for Hospital Hygiene at the DMHP) on the conceptual basis of Hospital Hygiene (HH), including the WASH (Environment, Water, Hygiene and Sanitation) component.

### Evaluation procedure

The process involved setting up a Regional Pilot Committee for the Control of Healthcare associated infections (CORELIN) in 2017, which organized statutory meetings with the heads of national technical structures involved in the issue. These stakeholders proceeded with a literature review on healthcare-associated infection control activities and the WHO trial evaluation framework, to develop an evaluation framework tool adapted to the Ivorian context. Hygiene monitoring tools developed by the DGS/MSHP and validated by all stakeholders were then made available to three UHCs and public health establishments in the Health Districts of Health Region Abidjan-2. Their application in the field required training for the focal points of these implementation structures by the team of experts at central level, on the concepts of healthcare-associated infections, standard precautions, and complementary measures. CORELIN includes the national experts, the Head of the Health Action Department of the Health Region Abidjan-2, the HH focal point of its 06 Health Districts, the Head of the HH Department of the University Hospital and the development partner.

Students from the first graduating class of the master’s degree in Hospital Hygiene and Safety from the Public Health Department of the Pharmaceutical and Biological Sciences Faculty were involved in the process to help them better grasp the basic elements of IPCAF. The outcome of the process was the endorsement by all stakeholders of the use of the contextualized IPCAF tool in the assessments of reference hospitals and ESPCs.

### Assessment framework tool

To strengthen the hygiene aspects, the WHO published in 2017 an assessment framework containing guidelines on the essential components of IPC programs to enable countries and health facilities to establish and strengthen their IPC activities. It contains eight essential components: (i) IPC program, (ii) IPC guidelines, (iii) IPC training and education, (iv) HCAI surveillance, (v) multimodal strategies, (vi) IPC practice monitoring/audit and feedback, (vii) workload, staffing, and bed occupancy, and (viii) IPC environments, materials, and equipment. The IPCAF framework includes individual questions on IPC aspects, and a score is assigned to each possible answer to a question. Thus, the framework is divided into eight sections following the concept of the eight essential components of IPC. The scores of the individual questions are aggregated for each essential component. A maximum score of 100 points can be achieved per individual question, and the highest possible overall IPCAF score is 800 points. The final IPCAF score is calculated by adding the scores of the eight core components and assigns an IPCAF level to each health facility assessed. Four IPC categories are possible for the overall score obtained: (inadequate) 0-200 points, (basic) 201–400 points, (intermediate) 401–600 points, and (advanced) 601–800 points [19, 23].

### Sampling

We selected 30 public health facilities in the Autonomous District of Abidjan through quota sampling, prioritizing those in the Health Districts of Koumassi-Port Bouët-Vridi and Abobo-Est as well as the eight general hospitals by reasoned choice.

### Assessment process and data collection technique

Activities for the HH and WASH infrastructure assessment began with stakeholder briefings and sensitization and training of two teams of five interviewers to harmonize all actors’ understanding of the assessment tool. The researchers are senior hygiene and sanitation technicians (6), sociologists (1), senior health managers (3) and come from the DHPSE (1), DMHP (1), Health Region Abidjan-2 (2), the Health Districts of Koumassi-Port Bouët-Vridi (1), Treichville-Marcory (1), Abobo-Est (1) and Cocody-Bingerville (1). Three (3) researchers were students on the master’s degree in Hospital Hygiene and Safety.

The Collection Tool was pretested and then administered from March 20–28, 2018, to gather the information needed to assess the 30 selected health facilities through interviews and observations during field visits.

The interviews were conducted with the IPC managers and staff of the evaluated facilities and focused on the organizational structure of an IPC program. The observation allowed us to assess the practices of the staff in the various departments visited. The observation focused on the operation of basic parameters in accordance with national norms and standards on hand hygiene, environmental and waste management. Photographs were taken as appropriate to illustrate the data from the interviews and observations. In addition, a document review of the sites visited provided information on the activities of the hygiene services.

The evaluation report was pre-validated with the Regional Pilot Committee for the Control of Healthcare associated infections (CORELIN). The final report on the results of the evaluation was coupled with the training of focal points on the IPC program. It was then shared with the technical and financial partner, the Ministry of Health, and the regional management to improve the planning of their hygiene activities.

### Methods of data analysis

A harmonization session of the collected data was conducted on April 12, 2018, and then a descriptive analysis was conducted for the Categories Data using frequency analysis and a cross-tabulation. Qualitative data from key informant interviews were grouped into meaningful patterns and/or themes through content and thematic analysis using NVivo software. In-depth analysis of each theme was undertaken using a three-step “describe, compare, connect” approach [24]. Individual interview data were linked to literature review data and institutional observations for a multidimensional description of the core components of each institution’s IPC. The analysis was based on the score values obtained, which specified the performance of the IPC program of the health-care facilities visited.

### Ethical considerations

The data used to write this article are part of the results of a Master 2 in Hospital Hygiene and Safety coordinated by the Department of Public Health of the UFR of Pharmaceutical and Biological Sciences of the Félix Houphouët-Boigny University of Abidjan.

Ethics approval was obtained from the *Comité Scientifique Interne Département de Santé Publique UFR des Sciences Pharmaceutiques et Biologiques Abidjan*, Côte d’Ivoire (N^o^ 165).

A written informed consent was obtained from all participants in the study. Participation was voluntary and participants were informed of their right to withdraw from the study when they wished to do so. All the participants were aware of the study’s purpose, risks, and benefits. Data were collected, managed, and analyzed in a way to ensure the confidentiality of study participants. All procedures performed in this study involving human participants were in accordance with the ethical standards of the national ethic review committee and with the 1964 Helsinki Declaration and its later amendments or comparable ethical standards.

## Results

The 30 health-care facilities assessed in this study included 8 HGs, 14 CSUCOMs, 5 FSUCOMs, 1 Municipal Hospital (MH), 1 SSSU and 1 CSR. Therefore, 8 intermediate-level facilities and 22 peripheral-level facilities of the Autonomous District of Abidjan were evaluated.

### Performance of health-care facilities in terms of ICP

An overall median IPCAF score of 242.5/800 was achieved for the 30 health-care facilities evaluated, which corresponded to an inadequate level at the collective level. When examining individual facility scores, the points ranged from 630 to 32.5. According to the WHO ICP level assignment, no facility achieved the “advanced” level of performance, 5 health-care facilities (17%) achieved the “intermediate” level, 10 (33%) were at the “basic” level, and 15 (50%) were at the “inadequate” level. Paradoxically, a CSU COM achieved the highest IPC score (630) ahead of the HGs. Only the HGs in the Health Districts of Abobo East, Abobo West, Anyama and Cocody-Bingerville obtained scores above 500. The other HGs had scores between 250 and 500 *(*Table 1*)*. Additionally, the organization of the IPC system in the Health Region Abidjan-2 was at a performance level of 17% for all intermediate level facilities.

**Table 1:** Summary of facility scores illustrating their IPC performance level

Health-care facilities evaluated		Score	Health-care facilities evaluated		Score
1	CSUCOM BANCO-SUD	630	16	CSUCOM AGUETO PK 18	225
2	HG ABOBO SUD	622.5	17	FSUCOM ABOBO-TE	197.5
3	HG ANYAMA	540	18	CSUCOM GONZAGUEVILLE	182.5
4	HG ABOBO NORD	535	19	SSSU	165
5	HG DE BINGERVILLE	517.5	20	CSUCOM QUARTIER DIVO	158
6	HG DE TREICHVILLE	392.5	21	CSU AKWABA D.WARF	155
7	HG DE PORT BOUËT	387.5	22	CSUCOM DJIBI	155
8	HG DE KOUMASSI	376.5	23	CSUCOM VRIDI 3	140
9	HG DE MARCORY	342.5	24	CSUCOM PANGOLIN	137.5
10	CSUCOM BOCABO	320.5	25	FSUCOM AVOCATIER	130
11	CSUCOM BC	305	26	CSUCOM AKLOMIABLA	112
12	CSUCOM KENNEDY	302	27	FSUCOM VRIDI CANAL	110
13	HM VRIDI CITE	295	28	CSUCOM CITE HB	110
14	FSUCOM AKEIKOI	270	29	CSUCOM ZOE BRUNO	80
15	FSUCOM ABOBO-BAOULE	260	30	CSR ADJAHUI COUBE	32.5

### Essential components of IPC programs evaluated in healthcare facilities

#### IPC Program

An IPC program includes a hygiene committee, a hygiene correspondent, and an action plan. Seventeen health-care facilities visited (56.7%) had a hygiene committee or correspondent, of which 14 (82.35%) had also developed an annual action plan. All 8 HGs had a hygiene committee and a well-structured action plan. Nine ESPC (41%) had a hygiene committee consisting of untrained hygiene staff. Only 2 (21%) of these ESPCs had an action plan.

#### Availability of guidelines

**Fig. 2:**
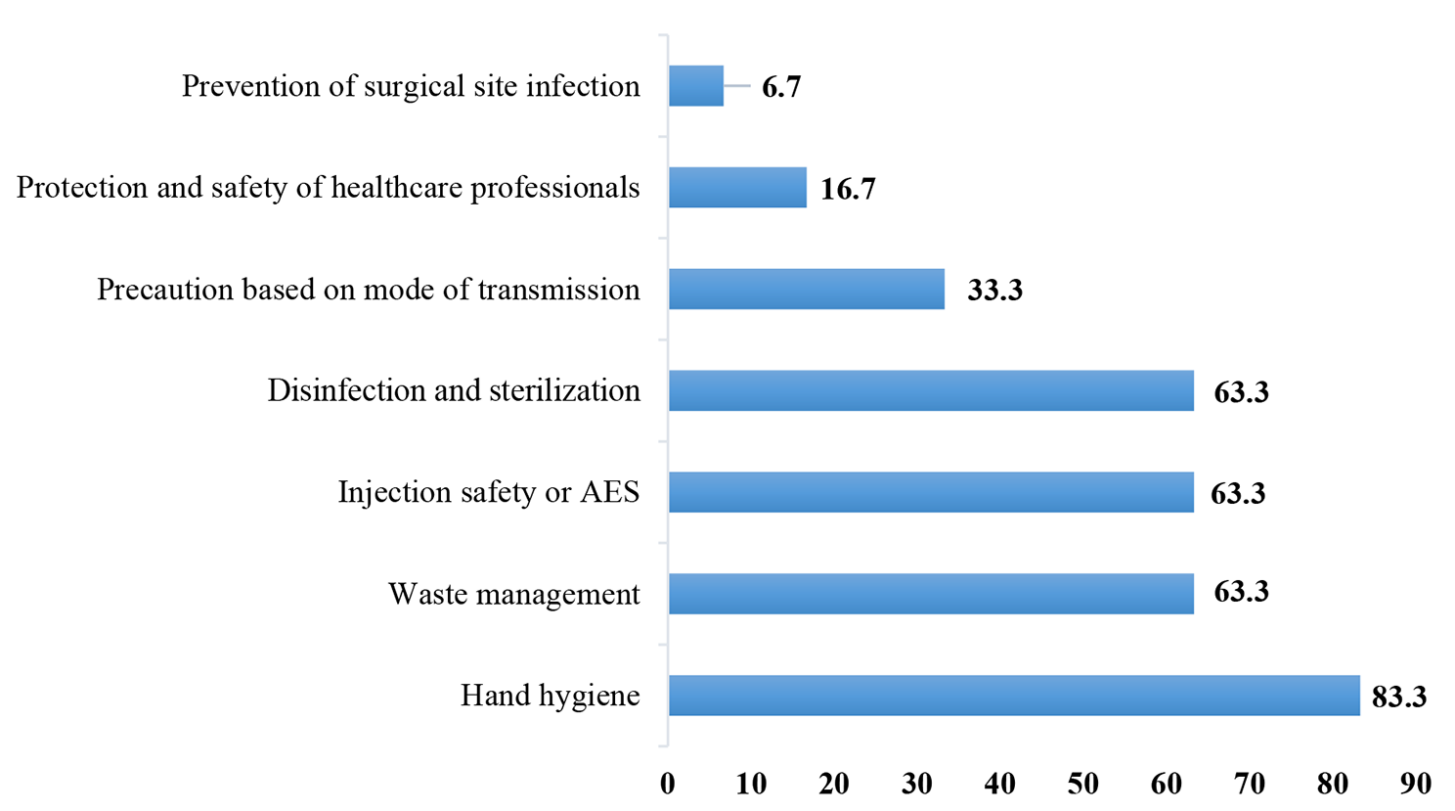
Availability of essential guidelines in health-care facilities

Eight items were addressed in terms of the availability of guidelines with thirteen subitems. Figure 2 summarizes seven subitems, of which three were unacceptable. The guidelines available in most of the facilities assessed were precautions based on national standards such as hand hygiene (83.3%), injection safety (63.3%), and sanitary waste management (63.3%). In contrast, staff involvement, staff training, and monitoring of guideline implementation were not *performed (*Fig. 2*).*

The six subitems not represented were prevention of hospital-acquired pneumonia, prevention of catheter-associated urinary tract infections, prevention of multidrug-resistant infections, and antibiotic stewardship. They were not included in any of the documents from the health-care facilities evaluated.

#### IPC training and education

Twelve facilities (40%) reported having at least one qualified resource person to provide IPC training, while 18 (60%) did not. The availability of an IPC trainer was generally an issue at the ESPC.

Twenty-one facilities (70%) had not trained either their staff or trainees in the past two years, while 7 (23.3%) reported having done so, and 2 (6.7%) reported having trained only their staff. Twenty-eight institutions evaluated (93.3%) did not evaluate the training process of their staff, except for one (3.3%), and it was not formal IPC training.

#### Monitoring and auditing IPC practices

**Fig. 3:**
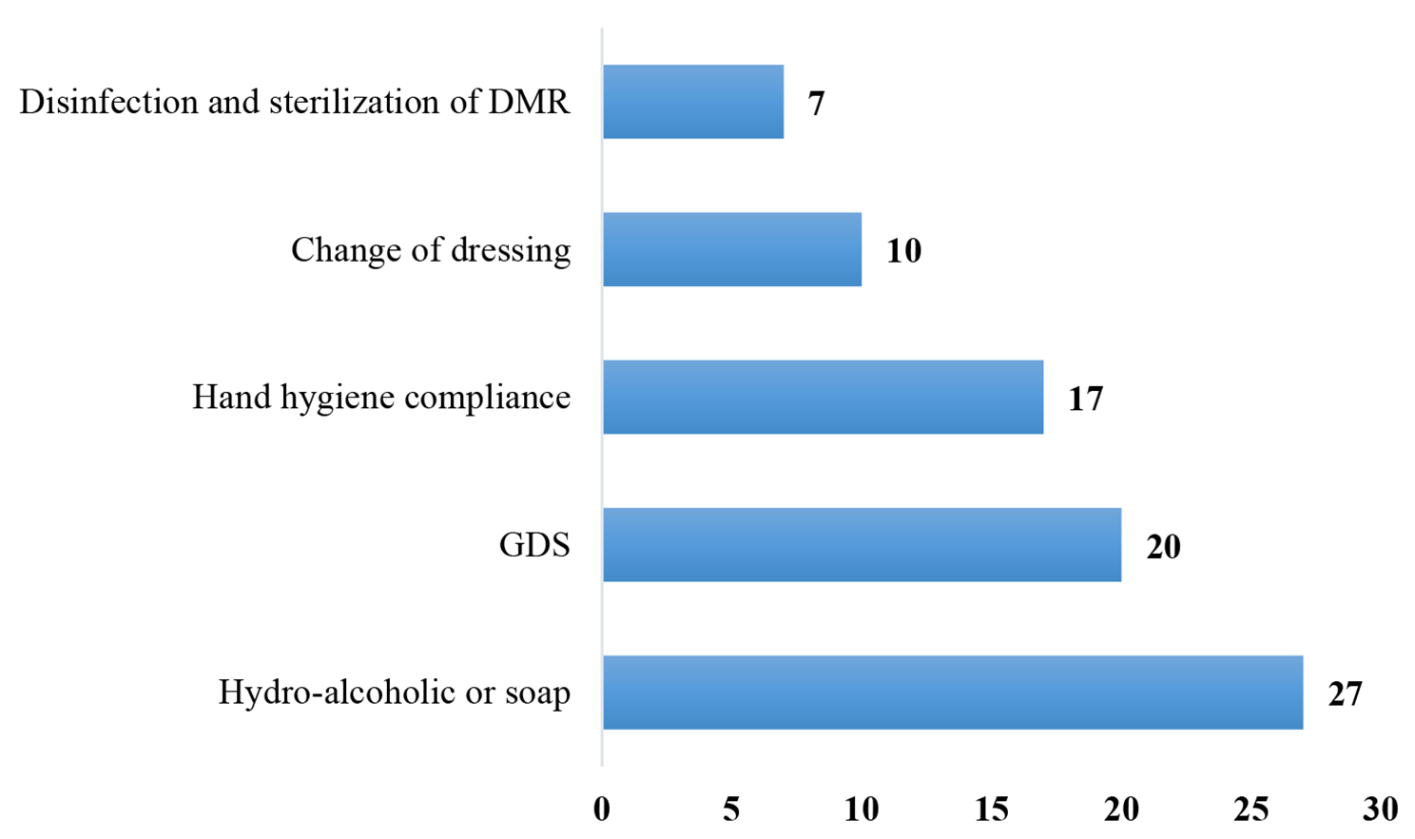
Proportion of health-care facilities by monitoring indicators

All the health-care facilities evaluated had unacceptable performance in monitoring and auditing IPC practices. There was no institutional culture in place to improve and change IPC behavior in these facilities *(*Fig. 3*).*

Nine health-care facilities (30%) had qualified staff responsible for monitoring/auditing, of which only 1 (3.3%) had a well-defined monitoring plan and 1 (3.3%) had developed an audit report on the status of HH activities.

### Environment, Equipment and materials, Water, Sanitation and Hygiene (WASH)

#### Potable water and wastewater

Twenty-eight health-care facilities (93.3%) used drinking water from the SODECI, and 2 health-care facilities (6.7%) in the communes of Koumassi and Port Bouët used water from traditional wells because of the problems of frequent untimely interruptions in the water supply from the SODECI. To remedy this, the health-care facilities stocked up on water using storage drums (polyethylene tanks) or used well water. However, no provision was made to guarantee the quality of water stored for more than two days. In addition, the storage drums and containers were not properly maintained. Health facilities with traditional wells did not treat the water before it was used for treatment.

Most of the wastewater from health care activities was discharged into septic tanks installed for this purpose. None of the health-care facilities visited had a wastewater treatment plant, yet the heavy metal content of the water was often very high.

#### Hand washing

Twenty-one health-care facilities (70%) had nonfunctional handwashing facilities (lack of drinking water and products), while 3 (10%) did not have any. Thus, hand hygiene compliance was not properly practiced at key times. Only 6 health-care facilities (20%) had a functional hand washing point. In addition, hand washing points donated by Ebola partners were out of order due to the quality of the plumbing materials.

#### Hygiene and sanitation

Some toilets in the facilities visited were functional (52%), defective (17%) or partially functional (31%). Most toilets (55%) were poorly maintained. There were various types of toilets, ranging from improved toilets with English seats or sanitary bowls to Turkish toilets and improved ventilated pit latrines. Some toilets were privately managed to maintain sustainable ownership. In general, manual flush toilets did not function properly due to their age. Some toilets had foul odors due to poor maintenance and lack of flushing. The upper surfaces of the defecation stalls were insufficiently cleaned.

#### Maintenance of the rooms

Only the Bingerville GH has a formalized process for cleaning the premises through a tool that tracks the cleaning of the premises and toilets. In general, the frequency of cleaning the premises was once or twice a day. The outside environment was cleaned every morning.

#### Sterilization of equipment

Three health-care facilities visited (10%) had a sterilization room or department for reusable devices, while 27 (90%) did not. Regarding the treatment of reusable medical devices (DMRs), disinfection was performed with highly diluted bleach and without traceability, and the bleach dilution protocols were not known by the staff. There is no disinfection room, so disinfection is performed in inappropriate rooms that may be close to the toilets or in stores with soaking tanks that are not graduated or covered. Medical devices (thermometers, blood pressure monitors, stethoscopes, etc.) were often not disinfected.

### Waste management

Ten health-care facilities visited (33.3%) had sufficiently functional and well-identified waste containers, 17 (56.7%) had unidentified or unbagged containers, and 3 (10%) had insufficient containers.

#### Method of waste elimination

Twenty-one establishments visited (70%) burned their waste in the open air, 5 (16.7%) entrusted it to a private service provider, i.e., a solid waste pre-collector, and 4 (13.3%) used an alternative incinerator or burner. None of the facilities had a functioning modern incinerator or a banalizer. Port Bouët GH had a modern 15 kg/H incinerator that had not been operational for two years.

The analysis of the observation and documentation data revealed the following: an absence of waste management tools, waste sorting was not done properly at the production site, waste collection equipment at the production site was insufficient and poorly adapted, waste garbage cans lacked appropriate waste bags, color codes were not respected in most facilities, there was no centralized waste storage space and the existing spaces were often inadequate; the final treatment of the waste was done in situ in the open air or with burners.

## Discussion

This study analyzes hospital hygiene and WASH infrastructure according to the components of the WHO IPCAF framework in 30 health-care facilities in the autonomous district of Abidjan, particularly in the Health Region Abidjan-2 in Cote d’Ivoire. It represents the first application of the IPCAF tool in this country. It highlighted the level of performance of the implementation of the IPC program in the evaluated facilities and raises issues that deserve to be discussed.

This study showed an overall median IPCAF score of 256.7/800, which corresponded to a basic level of the IPC program in all the health-care facilities visited. Most facilities were at the basic and inadequate levels of IPC at the individual level. The organization of IPC activities (especially for components 1, 2, and 3) was not always in place in almost all the facilities assessed. This greatly hindered the achievement of higher performance scores across all institutions. These results reflect the fact that HH structures and activities were not yet formally established in all Ivorian health facilities in accordance with Decree No. 98–379 of June 30, 1998, which stipulates the establishment of a hospital hygiene service in urban public health facilities that do not have the status of a National Public Institution.

Our overall IPCAF score is similar to those of IPC programs implemented in low-income countries reported by Tomczyk S et al. [25]. This is similar to that found by Opollo MS et al. (2021) at Lira Teaching Hospital in Uganda, quantified as 225/800 in terms of overall score, which refers to a basic level of IPC compliance [13]. This result is also consistent with the result of Rayson D et al. (2021) (median overall HH score, basic, of 212.5) in an assessment of 18 health facilities in the Mwanza region of Tanzania [11].

Our results in terms of IPC components are not isolated and limited to Ivorian institutions; they have been highlighted in other countries. Jeong Y et al. (2022) reported an IPC training rate of less than 30% in all hospital groups in the Republic of Korea, while the prevention of healthcare associated infections was low in primary- and intermediate-level hospitals (25%) [21]. An Australian study found weak education and training on IPC practices and limited implementation of IPC interventions [26]. Training was rarely or never provided in Uganda, while monitoring and follow-up/audit of IPC activities existed [13]. Oppong TB et al. (2020) found inadequate provision of detergents, running water and personal protective equipment (gloves, masks, gowns, etc.) in 56 Ghanaian acute care hospitals [18]. IPC guideline components were low in 32 hospitals in the Indian healthcare associated infections Surveillance Network [27]. The individual score for the component “monitoring/auditing of practices and IPC feedback” was lowest in 11 health facilities in eastern China [17]. Three health facilities in the Ashanti region of Ghana had gaps in antimicrobial stewardship capacity and compliance with WHO guidelines on IPC [9]. The core component “education and training” was among the lowest scores, but low-income countries had the lowest scores in the core components “surveillance of hospital-acquired infections” and “monitoring, auditing of IPC practices and feedback.“ In addition, the study showed limited access to functional toilets and hand hygiene stations at the point of care in low-income countries [25].

In contrast, our result lags far behind those of European, American and Asian countries [17, 21, 26, 27]. In neighboring Ghana, a study involving 56 surveyed facilities found that 19 had an IPC program with clearly defined objectives. Overall, 8 facilities had an advanced level of IPC readiness, 18 an intermediate level, 23 a basic level, and 7 an inadequate level [18]. Another study conducted in three Ghanaian hospitals found that they had basic and intermediate IPC systems with scores of 385, 487.5 and 435.8 out of 800 [9].

Developed countries have implemented key aspects of the IPC program and have achieved good levels of implementation through mostly advanced practices [26, 27]. These results can be explained by the contribution of existing legislation [19], the strong involvement of policy makers, and the implementation of the IPC budget [21]. Indeed, in 2011, the German Infection Protection Act (“Infektionsschutzgesetz”) was revised, increasing the importance of IPC in hospitals [19]. In 2018, the National Health Commission of the People’s Republic of China issued the “Accreditation Regulations of Hospital Infection Control and Prevention,“ which specifies the basic principles, assessment content, and requirements of IPC. For example, annual surveys on the prevalence of hospital-acquired infections have become mandatory in mainland China [17].

Further analysis of the different core components of the IPC program in our study yielded diverse results. Mid-level facilities excelled in implementing an IPC program with a hygiene committee and more structured operational plans. Paradoxically, a peripheral-level facility achieved the highest IPC score ahead of the HGs because the latter had rigorous management of its funding. The guidelines were adapted to national standards and were not optimal. Staff education and training on IPC practices was a major weakness in this study, with implications for staff involvement and implementation of the guidelines. The hygiene staff were often coached by the manager or chief medical officer who was not trained on the issue (no formal IPC training was given or received by staff). This explains the lack of planning of their activities. The commitment of the head of the institution was not strongly felt and suggested that the responsibilities of the actors would not be clearly defined. In addition, IPC training, committee meetings, monitoring and auditing activities were not regular, well-structured, or documented. The few existing reports were not properly archived. These findings strongly contributed to the poor results of this evaluation, with a lack of skills and unacceptable performance in the monitoring and auditing of IPC practices.

Prevention of healthcare associated infections and antibiotic management were not practiced in the evaluated facilities. This result impacts the quality of health services provided and the hospital case fatality and morbidity rates as well as the overall mortality rate [20].

Aspects of WASH (environment, water, hygiene, and sanitation) and waste management were either deficient, nonexistent, or severely limited in almost all the health facilities assessed. This suggests that these facilities did not yet have appropriate structural arrangements for the successful implementation of the IPC program as recommended by the WHO [8, 16, 18]. This result highlights the thorny challenge of sustaining achievements in Africa in general. Indeed, substantial resources were mobilized to prepare the response to the Ebola epidemic, but not all of the achievements of this fight have been sustainable [20].

The IPC and hand hygiene programs were designed by the WHO to facilitate the achievement of Universal Health Coverage (UHC) because they have a direct impact on the quality of care and patient safety at all levels of health services [16]. Nevertheless, we note from the various results obtained that the implementation of IPC components remains problematic, regardless of the level of development of the countries, and raises challenges identical to those of any intervention. It involves contextual aspects, adequate human, material and financial resources, behavioral characteristics, involvement of all stakeholders, specific strategies and expertise in IPC [8].

The lessons learned from the very recent global COVID-19 pandemic should convince the governments of the world, especially those of resource-limited countries, to make a sustained investment in IPC and hand hygiene to ensure better national health.

At the conclusion of this work, we recommend the following:

- IPC certification through the establishment of national infection control committees and networks as in other countries: Germany, Korea, China, etc. (European Committee and African Infection Control Network)
- the establishment of IPC training programs in Ivorian universities and training institutes.
- the availability of increased and regular funding (budgets with lines, state subsidies, partner support) dedicated to IPC.
- the development of specific expertise in IPC through training and strengthening of human resources in IPC.
- the development of a real sustainability plan for the sustainability and ownership of the achievements of technical and financial partners, as in the case of the COVID-19 pandemic.

### Study limitations

This study has some relevant limitations: multimodal strategies have not been developed because they are relatively new concepts that pose problems of understanding for stakeholders and are difficult to inform in our context of a limited-income country. The IPCAF is perceived as potentially compromising by the institutions evaluated and their managers. This is because it collects sensitive information on financial resources and their management, the overall functioning of the services, which gives information on the quality of the services provided, and the accountability of health workers. Therefore, in some cases, answers to the questions could be incorrect and deliberate to obtain a higher score. The same could be said for facilities that are very interested in the ISO certification aspects that are in vogue in national administrations and institutions. Uncorrected data entry errors are also possible.

## Conclusion

IPC structures and processes are very limited and in an embryonic state in Cote d’Ivoire for all components. This assessment provides opportunities for improvement in the implementation of the IPC program in our country by identifying the strengths and weaknesses of the program. It represents a state of the art and will serve as a basis for the implementation of robust IPC programs in all health facilities in the country. To do this, the government must show political will and strong leadership through strict regulations, provision of resources, development of IPC expertise and rigorous monitoring of the process. must reform its health system to achieve universal health coverage (UHC) with efficient health services for patient safety. This can only be achieved through the effective implementation of a sustainable IPC program in all health facilities and the involvement of all stakeholders.

## Data Availability

The datasets used and/or analyzed during the current study are available from the first author on reasonable request.

## Abbreviations

CORELIN: Comité Régional Pilote de Lutte contre les Infections Nosocomiales *(Regional Pilot Committee for the Fight against Healthcare associated infections)*
CSR: Centre de Santé Rural *(Rural Health Center)*
CSUCOM: Centre de Santé Urbain Communautaire *(Urban Community Health Center*)
DGA-HP: Directeur Général de Santé Adjoint chargé de l’Hygiène Hospitalière *(Deputy Director General of Health in charge of Hospital Hygiene)*
DGS: Direction Générale de la Santé *(General Health Directorate)*
DHPSE: Direction de l’Hygiène Publique et de Santé-Environnement *(Directorate of Public Hygiene and Health-Environment)*
DMHP: Direction de la Médecine Hospitalière et Proximité *(Department of Hospital Medicine and Proximity)*
DMR: Dispositifs Médicaux Réutilisables *(Reusable Medical Devices)*
ESPC: Établissements de Santé de Premier Contact *(First Contact Health Establishments)*
FSUCOM: Formation Sanitaire Urbaine Communautaire *(Urban Community Health Units)*
IPCAF: Infection Prevention Controle Assessment Framework
MSHP: Ministère de la Santé et l’Hygiène Publique *(Ministry of Health and Public Hygiene)*
SODECI: Société de Distribution d’Eau de Cote Ivoire *(Ivory Coast Water Distribution Company)*
SSSU: Service de Santé Scolaire et Universitaire *(School and University Health Service)*
UFR: Unité de Formation et Recherche *(Training and Research Unit)*
